# Inadequate gastric preparation and its associated factors for magnetically controlled capsule endoscopy

**DOI:** 10.3389/fphar.2023.1184754

**Published:** 2023-08-28

**Authors:** Qing-Zhou Kong, Cheng Peng, Zhen Li, Bao-Ling Tian, Yue-Yue Li, Fei-Xue Chen, Xiu-Li Zuo, Yan-Qing Li

**Affiliations:** ^1^ Department of Gastroenterology, Qilu Hospital, Shandong University, Jinan, Shandong, China; ^2^ Shandong Provincial Clinical Research Center for Digestive Disease, Jinan, Shandong, China; ^3^ Robot Engineering Laboratory for Precise Diagnosis and Therapy of GI Tumor, Qilu Hospital, Shandong University, Jinan, Shandong, China

**Keywords:** magnetically controlled capsule endoscopy, gastric disease, preparation, proton pump inhibitor, simethicone

## Abstract

**Goals:** To explore factors associated with inadequate gastric preparation for MCE.

**Background:** Factors associated with inadequate gastric preparation for magnetically controlled capsule endoscopy (MCE) remains unclear.

**Study:** Data of patients who underwent MCE from June 2021 to July 2022 were prospectively collected. The gastric cleanliness score (GCS) of the six stomach regions (gastric cardia, fundus, body, angulus, antrum, and pylorus) was recorded. Patients with GCS score ≥18 were defined as the adequate preparation. Factors related to inadequate gastric preparation were analyzed using a logistic regression model with estimated odds ratios (OR).

**Results:** The mean GCS score of 211 patients was 17.01 ± 2.82. In the multivariable analysis, proton pump inhibitor (PPI) use (OR 3.57; 95% CI 1.69–7.95; *p* < 0.01) and premedication time after administering simethicone <30 min (OR 2.86; 95% CI 1.10–7.39; *p* = 0.03) were independent risk factors for inadequate gastric preparation. Comparing the gastric cleanliness of different locations, the median GCS of the lower stomach [10.00, IQR (9.50, 11.00)] was significantly higher than that of the upper stomach [7.00, IQR (6.00, 8.00)] (*p* <0.001).

**Conclusion:** PPI use and inadequate premedication time (<30 min) may reduce the quality of gastric preparation for MCE. The type, dose, duration of medication, and discontinuation time of PPIs was well worth further exploration. Appropriate control of the type and time of premedication may be the key to improving overall gastric cleanliness.

## 1 Introduction

Magnetically controlled capsule endoscopy (MCE) offers a safe means to screen the gastric mucosa, detect lesions and identify gastric cancer, using a remote magnetic control system to adjust the angle of capsule observation without the need for intubation or sedation (Keller et al., 2011; Liao et al., 2012; Van Cutsem et al., 2016; Kim et al., 2018; Zhang et al., 2018). Multiple large, prospective clinical studies have shown that MCE has considerable diagnostic efficacy in examining gastric lesions compared to gastroscopy (Rey et al., 2012; Zou et al., 2015; Liao et al., 2016; Geropoulos et al., 2021; Li et al., 2021). Due to its minimal invasiveness and lack of sedation requirements, MCE has gained widespread acceptance as a diagnostic modality (Eliakim, 2017; Jiang et al., 2022).

The diagnostic performance of MCE relies on clear visualization of the entire gastric mucosa. However, in clinical practice, the presence of chyme, air bubbles, mucus, and bile in the gastric lumen may obscure microscopic lesions, reduce the cleanliness of the gastric mucosa, compromise the integrity of the visual field, and potentially lead to misdiagnosis or omission (Zhu et al., 2018). Unlike traditional gastroscopy, MCE does not provide the ability to clean the gastric mucosa by spraying water. Therefore, the quality of gastric preparation in MCE greatly affects the diagnostic accuracy for gastric lesions.

Previous studies have shown that simethicone use can improve gastric image quality by removing air bubbles (Chang et al., 2014; Zhu et al., 2018), and repetitive positional changes may improve gastric cleanliness (Wang et al., 2019). Nevertheless, no systematic evaluation of factors influencing the quality of gastric preparation for MCE has been conducted. In this prospective observational study, our objective is to explore these factors and provide valuable insights into pre-examination preparation for MCE in clinical practice.

## 2 Materials and methods

### 2.1 Patients

This prospective observational study was approved by the Ethics Committee of Qilu Hospital, Shandong University and registered at ClinicalTrials.gov (NCT04933643). We included patients aged 18–75 years who required MCE at the endoscopy center of Qilu Hospital, Shandong University, from June 2021 to July 2022. All patients provided written informed consent.

Patients with the following were excluded: 1) severe physical diseases who were unable to adhere to the examination requirements; 2) dysphagia, known or suspected gastrointestinal fistula, stenosis or obstruction; 3) known active upper gastrointestinal bleeding; 4) altered gastrointestinal anatomy due to previous surgery; 5) exclusion criteria for magnetic resonance imaging examinations, including patients with implanted electronic medical instruments and magnetic metal devices; 6) pregnancy; and 7) claustrophobia or other mental disorders.

### 2.2 Gastric preparation protocol and MCE procedure

Patients were instructed to follow a soft diet the day before the examination and fast overnight (>8 h). Colored drinks were not permitted after 8 p.m. To reduce the impact of foam on the visual field, an appropriate bubble-removing agent (10 mL of simethicone dissolved in 50 mL of water) was administered 40 min before the examination. Before undergoing MCE, the patients were instructed to drink 500–1,000 mL of water to fill the stomach and provide the airwater interface for capsule sailing (Chinese Digestive Endoscopist Committee et al., 2017; Zhu et al., 2018; Capsule Endoscopy Group of the Chinese Society of Digestive Endoscopy, 2021).

For the MCE procedure, the patient ingested the capsule with 100 mL of water and was instructed to lay on the examination bed in the supine position. The operator then adjusted the endoscopic capsule using the magnetic control system. In order to observe the cardia, fundus, body, angulus, antrum, and pylorus in sequence, the patient position was adjusted as necessary. The patient position alternated between the left lateral, supine, and right lateral positions. At least two examinations of each gastric area were performed. The patient continuously consumed water when the stomach was underfilled. The gastric examination time for MCE was recorded.

### 2.3 Data collection

The content of the questionnaire, with the aim to record patient information and evaluate gastric cleanliness, was discussed and formulated jointly by the research team. Considerations included the relevant literature and available information, including patients’ demographic and related clinical data, such as patient sources, history of basic diseases (e.g., diabetes, hypertension), family history of gastric cancer, *H. pylori* infection, drug use, diet before examination, fasting time, and premedication time after administering simethicone. Premedication time after administering simethicone was defined as the time from administering simethicone to swallowing capsule.

### 2.4 Primary outcome

**FIGURE 1:**
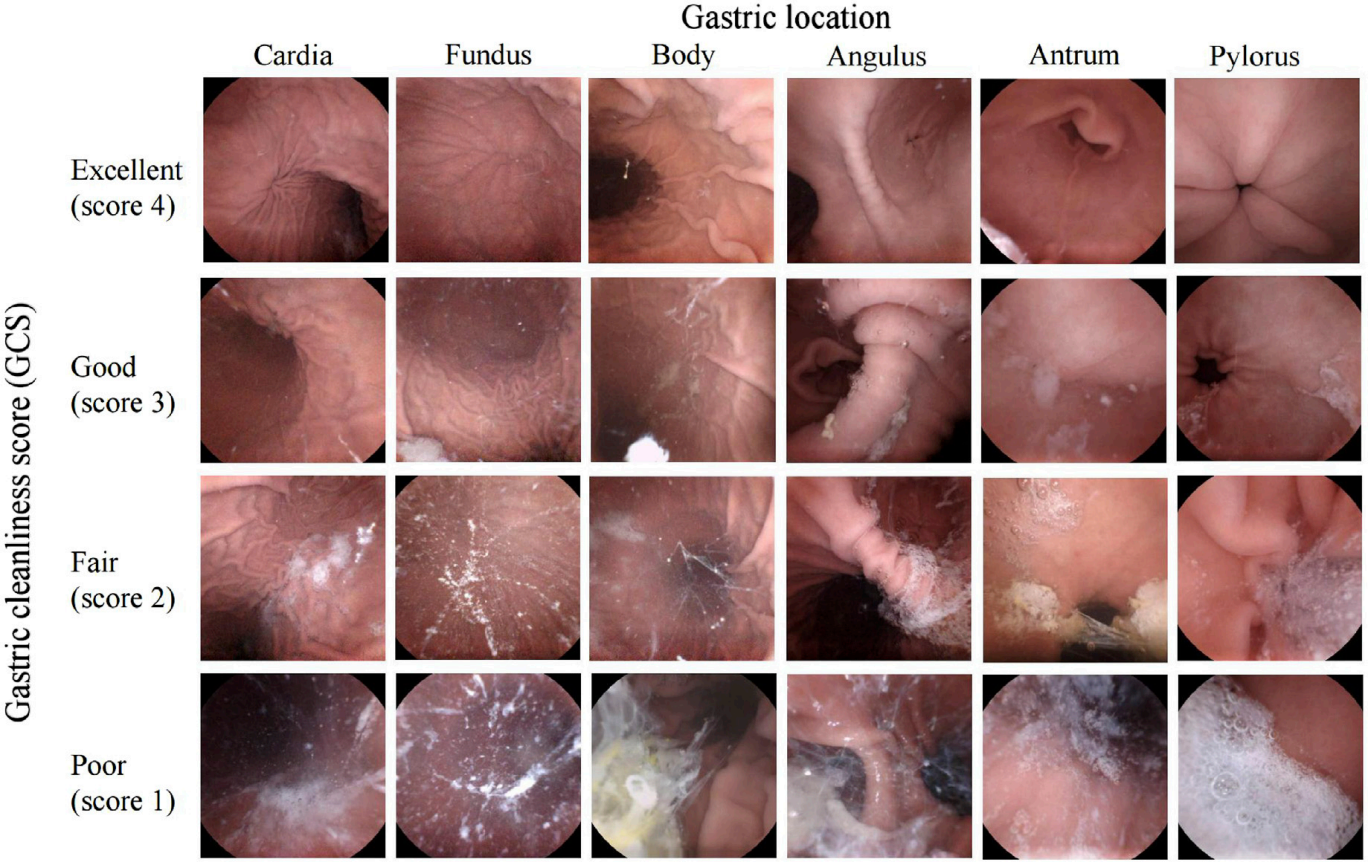
The 4-point grading scales for the gastric cleanliness of six gastric landmarks in MCE.

The primary outcome was to identify factors associated with inadequate gastric preparation. The gastric preparation quality was expressed as the gastric cleanliness score (GCS). We evaluated the six primary anatomical landmarks of the stomach (cardia, fundus, body, angulus, antrum, and pylorus). A 4-point grading scale was used to objectively assess the cleanliness of each landmark as either excellent (only traces of adherent mucus or foam present: score 4), good (small amount of mucus or foam present but no interference of the examination: score 3), fair (considerable amount of mucus or foam present preventing a completely reliable examination: score 2), or poor (large amount of mucus, foam, or chyme present that seriously impede observation: score 1) (Liao et al., 2016; Zhu et al., 2018). Since the gastric lumen is not sufficiently extended in the fasting state, quality was assessed on the basis of images obtained from each site sufficiently distended by water. The GCS was calculated as the total score of all six landmarks, ranging from 6 (totally inadequate) to 24 (perfect). A GCS ≥18 was considered adequate gastric preparation (Wang et al., 2019) (Figure 1), and these patients were defined as the adequate preparation group, while the remaining patients were defined as the inadequate gastric preparation group.

Using the scoring criteria, two endoscopists (with at least 3 years of reading experience) independently evaluated the cleanliness of the endoscopic images obtained during MCE. When the grading scores varied between the two endoscopists, the final GCS was determined by a senior endoscopist (with over 5 years of reading experience) who was in charge of quality control.

### 2.5 Secondary outcome

Secondary outcomes include upper and lower GCS and positive lesions. The senior endoscopist described and recorded any positive lesions observed during MCE. In this study, positive lesions were defined as focal lesions of the stomach, including focal erosion, polyps, ulcers, gastric varices, submucosal tumors, etc. Diffuse lesions, including superficial or atrophic gastritis, were regarded as negative. The number of lesions per patient (NLPP) was recorded to evaluate the total detection in the population.

### 2.6 Statistical analysis

Continuous variables are reported as means and standard deviation (SD), or medians and interquartile range (IQR) when not normally distributed, while categorical variables are expressed as percentages. Variables were compared using the chi-square or Kruskal–Wallis tests, when appropriate. Multivariable logistic regression analysis was performed to identify variables independently associated with inadequate gastric preparation quality, using estimated odds ratios (ORs) and 95% confidence intervals (95% CIs). Statistical significance was defined as a two-sided *p*-value of <0.05.

The sample size was calculated using events per variable (EPV), which is widely recognized as an effective means of determining sample size when the aim is to develop logistic regression prediction models (Wynants et al., 2015). According to the previous study (Wang et al., 2019), the proportion of the primary outcome (inadequate gastric preparation rate) was approximately 30% when the EPV was set at ten. Thus, to ensure reliability, at least 200 patients had to be enrolled in this study. In the cases where the univariate analysis showed results of *p* <0.10, multivariate analysis was performed. All statistical data were analyzed using R Statistics software (version 4.1.0, R Foundation for Statistical Computing, Vienna, Austria).

## 3 Results

### 3.1 Patients

Overall, 214 patients underwent gastric preparation and MCE from June 2021 to July 2022. Three patients were excluded due to upper gastrointestinal bleeding or missing data. Ultimately, 211 patients were enrolled in this study. The patient characteristics, clinical indications for MCE, and comorbidities are shown in Table 1.

**TABLE 1:** Demographic characteristics.

Characteristics	Overall cases (*n* = 211)	Gastric preparation of MCE		*p*-Value
		Inadequate (*n* = 114)	Adequate (*n* = 97)	
Age [median (IQR)]	53.00 (41.00, 64.00)	54.00 (44.25, 65.00)	51.00 (39.00, 63.00)	0.091
BMI [mean (SD)]	23.52 (3.43)	23.76 (3.21)	23.23 (3.68)	0.261
Male (%)	98 (46.4)	58 (50.9)	40 (41.2)	0.207
Education (%)				0.449
College or above	64 (30.3)	31 (27.2)	33 (34.0)	
Senior high school	38 (18.0)	21 (18.4)	17 (17.5)	
Junior high school	60 (28.4)	31 (27.2)	29 (29.9)	
Elementary or below	49 (23.2)	31 (27.2)	18 (18.6)	
Hospitalization (%)	24 (11.4)	17 (14.9)	7 (7.2)	0.124
Indication (%)				0.023
Abdominal pain	111 (52.6)	57 (50.0)	54 (55.7)	
Abdominal distension	31 (14.7)	15 (13.2)	16 (16.5)	
Other (nausea or acid reflux)	21 (10.0)	18 (15.8)	3 (3.1)	
Examination	48 (22.7)	24 (21.1)	24 (24.7)	
Family history of gastric cancer (%)	14 (6.6)	7 (6.1)	7 (7.2)	0.972
Smoking (%)	31 (14.7)	18 (15.8)	13 (13.4)	0.769
Drinking (%)	35 (16.6)	21 (18.4)	14 (14.4)	0.555
History of, No. (%)				
Diabetes mellitus	23 (10.9)	15 (13.2)	8 (8.2)	0.358
Hypertension	46 (21.8)	31 (27.2)	15 (15.5)	0.059
Coronary disease	34 (16.1)	22 (19.3)	12 (12.4)	0.240
NSAIDs use (%)	24 (11.4)	14 (12.3)	10 (10.4)	0.837
Statin use (%)	16 (7.6)	11 (9.6)	5 (5.2)	0.343
PPI use (%)	56 (26.5)	44 (38.6)	12 (12.4)	<0.001

MCE, magnetically controlled capsule endoscopy; IQR, interquartile range; BMI, body mass index; SD, standard deviation; NSAIDs, Non-steroidal anti-inflammatory drugs; PPI, proton pump inhibitor.

### 3.2 Inter-observer consistency assessment

High inter-observer consistency was found between the endoscopists in assessing the quality of gastric preparation. The intra-group correlation coefficient for the GCS was 0.89 (*p* <0.001) and the consistency for assessing the eligibility of gastric preparation between the assessors was ranked as “excellent” (Kappa = 0.914, *p* <0.001).

### 3.3 Primary outcome

Inadequate gastric preparation was reported in 54% (114/211) of the patients and the GCS was 17.01 ± 2.82 for the total population. Univariate analysis revealed that proton pump inhibitor (PPI) use (OR 4.45; 95% CI 2.18–9.08; *p* <0.001), nausea or acid reflux as an indication of MCE (OR 6.00; 95% CI 1.56–23.06; *p* = 0.01), hypertension (OR 2.04; 95% CI 1.03–4.06; *p* = 0.04), and premedication time after administering simethicone <30 min (OR 2.86; 95%CI 1.10–7.39; *p* = 0.03) were associated with inadequate gastric preparation.

In the multivariable analysis, PPI use (OR 3.57; 95% CI 1.69–7.95; *p* <0.01) and premedication time after administering simethicone <30 min (OR 2.86; 95%CI 1.10–7.39; *p* = 0.03) were independent risk factors for inadequate gastric preparation in patients requiring MCE (Table 2).

**TABLE 2:** Univariate and multivariable analysis of risk factors for inadequate gastric preparation of MCE.

	Univariate analysis		Multivariable analysis	
Characteristics	OR (95% CI)	*p*-value	OR (95% CI)	*p*-value
Male	1.48 (0.85–2.55)	0.16		
Age ≥65	1.84 (0.95–3.57)	0.07	1.52 (0.70–3.36)	0.29
BMI	1.05 (0.97–1.13)	0.26		
Hospitalization	2.25 (0.89–5.69)	0.09	1.83 (0.66–5.37)	0.25
Education				
College or above	1			
Senior high school	1.31 (0.59–2.94)	0.51		
Junior high school	1.14 (0.56–2.30)	0.72		
Elementary or below	1.83 (0.86–3.92)	0.12		
Indication of MCE				
Examination	1		1	
Abdominal pain	1.06 (0.54–2.08)	0.88	1.16 (0.70–3.36)	0.72
Abdominal distension	0.94 (0.38–2.31)	0.89	0.81 (0.30–2.20)	0.68
Other (nausea or acid reflux)	6.00 (1.56–23.06)	0.01	3.94 (0.97–20.46)	0.07
Smoking	1.21 (0.56–2.62)	0.63		
Drinking	1.34 (0.64–2.80)	0.44		
Family history of gastric cancer	0.84 (0.28–2.49)	0.75		
History of,				
Diabetes mellitus	1.69 (0.68–4.16)	0.26		
Hypertension	2.04 (1.03–4.06)	0.04	1.55 (0.70–3.48)	0.28
Coronary disease	1.69 (0.79–3.63)	0.18		
NSAIDs use	1.20 (0.51–2.85)	0.67		
Statins use	1.94 (0.65–5.80)	0.23		
PPI use	4.45 (2.18–9.08)	<0.001	3.57 (1.69–7.95)	<0.01
Fasting time	1.03 (0.96–1.12)	0.42		
Dinner the day before examination				
Fasting	1			
Liquid diet	0.86 (0.45–1.66)	0.62		
Non-liquid diet	1.36 (0.58–3.21)	0.48		
Premedication time after administering Simethicone				
≥40 min	1		1	
30–40 min	1.09 (0.61–1.95)	0.76	0.96 (0.51–1.82)	0.91
<30 min	2.86 (1.10–7.39)	0.03	3.81 (1.44–11.06)	0.01

MCE, magnetically controlled capsule endoscopy; BMI, body mass index; NSAIDs, Non-steroidal anti-inflammatory drugs; PPI, proton pump inhibitor.

### 3.4 Secondary outcome

**FIGURE 2:**
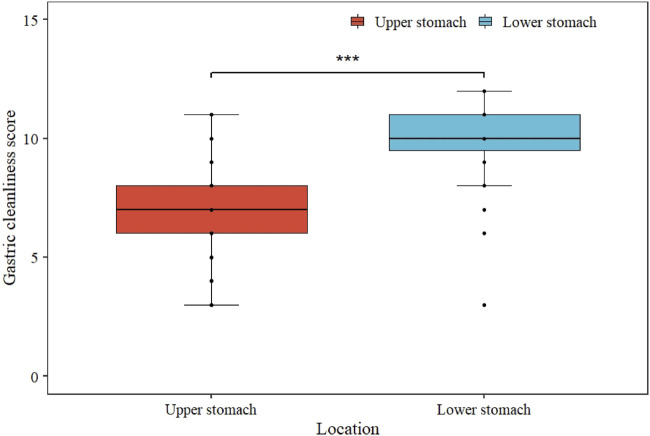
Comparison of upper and lower gastric cleanliness score. The median GCS of lower stomach was significantly higher than that of upper stomach (*** represents *p* <0.001).

Regarding the GCS of each site, the score of the lower stomach (the angulus, antrum, and pylorus) was higher than that of the upper (the cardia, fundus, and body), and the difference was statistically significant (*p* <0.001, Figure 2). Therefore, we opted to analyze the relationship between gastric preparation factors and upper gastric cleanliness. The multivariable analysis revealed PPI use (OR 4.01; 95% CI 1.91–8.88; *p* <0.001) and premedication time after administering simethicone <30 min (OR 3.60; 95% CI 1.37–10.41; *p* = 0.012) were associated with inadequate gastric cleanliness of the upper gastric site (Table 3).

**TABLE 3:** Univariate and multivariable analysis of risk factors for inadequate upper gastric preparation of MCE.

Characteristics	Univariate analysis		Multivariable analysis	
	OR (95% CI)	*p*-Value	OR (95% CI)	*p*-Value
Male	0.71 (0.37–1.35)	0.29		
Age ≥65	1.15 (0.53–2.53)	0.72		
BMI	0.94 (0.86–1.03)	0.20		
Hospitalization	2.15 (0.61–7.56)	0.23		
Education				
College or above	1			
Senior high school	0.54 (0.22–1.32)	0.18		
Junior high school	0.84 (0.37–1.93)	0.68		
Elementary or below	2.46 (0.82–7.39)	0.11		
Indication of MCE				
Examination	1		1	
Abdominal pain	1.16 (0.53–2.50)	0.71	0.88 (0.43–1.81)	0.73
Abdominal distension	1.55 (0.52–4.63)	0.43	0.69 (0.26–1.81)	0.46
Other (nausea or acid reflux)	7.43 (0.90–61.07)	0.06	3.40 (0.88–17.04)	0.10
Smoking	0.65 (0.28–1.54)	0.33		
Drinking	0.67 (0.29–1.51)	0.33		
Family history of gastric cancer	0.49 (0.16–1.53)	0.22		
History of,				
Diabetes mellitus	1.41 (0.45–4.36)	0.55		
Hypertension	1.23 (0.55–2.78)	0.62		
Coronary disease	1.41 (0.54–3.63)	0.48		
NSAIDs use	1.11 (0.39–3.15)	0.85		
Statins use	1.27 (0.35–4.66)	0.72		
PPI use	2.02 (0.88–4.63)	0.10	3.95 (1.90–8.67)	<0.001
Fasting time	1.04 (0.94–1.14)	0.46		
Dinner the day before examination				
Fasting	1			
Liquid diet	0.73 (0.35–1.54)	0.41		
Non-liquid diet	2.19 (0.62–7.80)	0.23		
Premedication time after administering Simethicone				
≥40 min	1		1	
30–40 min	1.27 (0.65–2.49)	0.49	0.97 (0.51–1.81)	0.91
<30 min	4.53 (1.00–20.53)	0.05	3.43 (1.32–9.75)	0.01

MCE, magnetically controlled capsule endoscopy; BMI, body mass index; NSAIDs, Non-steroidal anti-inflammatory drugs; PPI, proton pump inhibitor.

Positive lesions detected during MCE are shown in Table 4. There was a significant difference in the detection rate of positive findings between the two groups (*p* = 0.001). The NLPP in the adequate preparation group (0.77 ± 0.86) was significantly higher than that of the inadequate group (0.38 ± 0.83) (*p* <0.001). The NLPP for the lower stomach in the adequate preparation group (0.35 ± 0.50) was also higher than that of the inadequate group (0.14 ± 0.37) (*p* = 0.001), while there was no significant difference in the upper stomach comparison (*p* = 0.084).

**TABLE 4:** Gastric focal lesions detected by MCE.

Type of lesions	Overall cases (*n* = 211)	Gastric preparation of MCE		*p*-Value
		Inadequate (*n* = 114)	Adequate (*n* = 97)	
Focal erosion (%)	46 (21.8)	12 (10.5)	34 (35.1)	<0.001
Gastric polyps (%)	30 (14.2)	15 (13.2)	15 (15.5)	0.779
Gastric ulcers (%)	3 (1.4)	3 (2.6)	0 (0.0)	0.305
Others (%)	14 (6.6)	5 (4.4)	9 (9.3)	0.252
Total (%)	88 (41.7)	32 (28.1)	56 (57.7)	<0.001

MCE, magnetically controlled capsule endoscopy; Others include gastric varices, submucosal tumors, angiotelectasis, etc.

## 4 Discussion

Gastric cleanliness greatly affects the quality of MCE, and the presence of gastric mucus and foam reduces clinical observation ability (Rahman et al., 2016; Zhu et al., 2018). In able to ensure accurate MCE, it is crucial to systematically explore the factors that affect the quality of gastric preparation. We prospectively collected and analyzed factors associated with the quality of MCE gastric preparation. Logistic regression analysis indicated that PPI use and inadequate premedication time after administering simethicone affected gastric preparation quality.

PPI use as an independent risk factor for reduced gastric mucosal cleanliness may be associated with its impact on gastric emptying (Anjiki et al., 2005; Takahashi et al., 2006; Sanaka et al., 2007; Sanaka et al., 2010; Li et al., 2020). The “acid-pepsin maldigestion hypothesis” proposes that PPI use reduces pepsin activity and hinders the hydrolysis process by elevating the gastric pH, and thus prolonging the persistence of undigested large particles in the stomach. Ota et al. (2021) suggested that suppression of gastric acid secretion delayed gastric fluid emptying, which may be related to elevated levels of ghrelin and abnormal gastric peristalsis (Parkman et al., 1998; Sanaka et al., 2010).

As for the drugs used for gastric preparation, previous studies have shown that simethicone, an antifoaming substance, can effectively improve the visibility of the gastric mucosa (Chang et al., 2014; Elvas et al., 2017; Zhu et al., 2018). Regarding upper gastrointestinal endoscopy, Chang et al. (2014) suggested that 5 mL of simethicone suspension administered >30 min before the exam provides clear endoscopic visibility, while Woo et al. (2013) recommended a premedication time of 10–30 min. However, the optimal premedication time for MCE remains unknown. In some studies the medication was administered 50 min or 1 h before the examination (Keller et al., 2011; Rey et al., 2012), while 40 min is recommended by the consensus (Chinese Digestive Endoscopist Committee et al., 2017; Capsule Endoscopy Group of the Chinese Society of Digestive Endoscopy, 2021). In our study, premedication time <30 min after administering the simethicone resulted in substandard gastric mucosal cleanliness, while there was no differential gastric preparation quality between the 30–40 min and >40 min intervals. Simethicone dissolved in 50 mL of water will be emptied after antifoaming sufficiently. However, during MCE, the patient is required to consume a large amount of water before swallowing the capsule to provide an airwater interface. If the interval between medication administration and the initiation of water consumption is too short, gastric emptying and simethicone removal can be accelerated, and the contact and action time of simethicone and gastric mucosa will be insufficient, resulting in inadequate gastric cleanliness.

Similar to previous studies (Zou et al., 2015; Rahman et al., 2016; Wang et al., 2019), we found that the mucosal cleanliness of the upper stomach was worse than that of the lower stomach. Due to the effects of gravity and the left lateral and supine positions used during MCE, mucus accumulates more easily at the gastric fundus than at the antrum and pylorus. The effects of gravity also decrease the mucosa-detergent contact time in these regions, which again reduce the quality of gastric preparation. Further studies are warranted to explore potential drugs or methods specifically targeting upper gastric cleanliness.

This study had some limitations. The data were derived from a single center and may have some bias in patient characteristics. Further validation in multicenter studies is desirable. Second, PPI use was found to potentially reduce the quality of gastric preparation for MCE. However, the type, dose, duration of medication, and discontinuation time of PPIs were not collected and analyzed because of the limited sample size, and future studies might consider including this data. Other types of gastric acid secretion inhibitor should also be studied in future. Although pronase and other mucolytics have been reported to be effective in conventional gastroscopy (Bhandari et al., 2010; Liu et al., 2018), their use did not show significant improvement in MCE compared to the use of simethicone alone (Chang et al., 2014; Zhu et al., 2018). Therefore, pronase was not used in our protocol. Further studies are needed to explore the effect of pronase for MCE and determine the optimum dose required to maximize the mucolytic action of pronase. In addition, *H. pylori* infection was not included in the final analysis due to the restriction of urea breath test in some patients taking PPIs (Malfertheiner et al., 2022). Further methods (e.g., gastroscopy) could be used in prospective studies to assess the effect of *H. pylori* infection on gastric cleanliness.

In conclusion, our study showed that PPI use may reduce the quality of gastric preparation for MCE, whereas adequate premedication time (≥30 min) after administering simethicone may improve the cleanliness of the gastric mucosa. The type, dose, duration of medication, and discontinuation time of PPIs was well worth further exploration. Improving the type and time of premedication may be the key to improving the overall gastric cleanliness. Better control of these factors in the future will improve gastric cleanliness and a higher lesion detection rate is expected, which will be conducive to promoting the application of MCE in gastric cancer screening.

## Data Availability

The original contributions presented in the study are included in the article/supplementary material, further inquiries can be directed to the corresponding author.
